# Smart Glasses for Supporting Distributed Care Work: Systematic Review

**DOI:** 10.2196/44161

**Published:** 2023-02-28

**Authors:** Zhan Zhang, Enze Bai, Karen Joy, Partth Naressh Ghelaa, Kathleen Adelgais, Mustafa Ozkaynak

**Affiliations:** 1 School of Computer Science and Information Systems Pace University New York, NY United States; 2 School of Medicine University of Colorado Aurora, CO United States; 3 College of Nursing University of Colorado Aurora, CO United States

**Keywords:** smart glass, care coordination, telemedicine, distributed teamwork, mobile phone

## Abstract

**Background:**

Over the past 2 decades, various desktop and mobile telemedicine systems have been developed to support communication and care coordination among distributed medical teams. However, in the hands-busy care environment, such technologies could become cumbersome because they require medical professionals to manually operate them. Smart glasses have been gaining momentum because of their advantages in enabling hands-free operation and see-what-I-see video-based consultation. Previous research has tested this novel technology in different health care settings.

**Objective:**

The aim of this study was to review how smart glasses were designed, used, and evaluated as a telemedicine tool to support distributed care coordination and communication, as well as highlight the potential benefits and limitations regarding medical professionals’ use of smart glasses in practice.

**Methods:**

We conducted a literature search in 6 databases that cover research within both health care and computer science domains. We used the PRISMA (Preferred Reporting Items for Systematic Reviews and Meta-Analyses) methodology to review articles. A total of 5865 articles were retrieved and screened by 3 researchers, with 21 (0.36%) articles included for in-depth analysis.

**Results:**

All of the reviewed articles (21/21, 100%) used off-the-shelf smart glass device and videoconferencing software, which had a high level of technology readiness for real-world use and deployment in care settings. The common system features used and evaluated in these studies included video and audio streaming, annotation, augmented reality, and hands-free interactions. These studies focused on evaluating the technical feasibility, effectiveness, and user experience of smart glasses. Although the smart glass technology has demonstrated numerous benefits and high levels of user acceptance, the reviewed studies noted a variety of barriers to successful adoption of this novel technology in actual care settings, including technical limitations, human factors and ergonomics, privacy and security issues, and organizational challenges.

**Conclusions:**

User-centered system design, improved hardware performance, and software reliability are needed to realize the potential of smart glasses. More research is needed to examine and evaluate medical professionals’ needs, preferences, and perceptions, as well as elucidate how smart glasses affect the clinical workflow in complex care environments. Our findings inform the design, implementation, and evaluation of smart glasses that will improve organizational and patient outcomes.

## Introduction

### Background

Effective and timely care coordination and communication are critical components of efficient and safe patient care [1,2]. Failure in providing coordinated care and communicating patient data is seen as one of the root causes of adverse events such as delays in patient care and deviations from standard medical procedures [3]. The challenges in maintaining effective care coordination and communication are exacerbated when care providers are distributed (eg, located in different places) [4,5]. Over the past 2 decades, many telemedicine systems have been developed to augment remote clinical consults [6-8]. During the COVID-19 pandemic, the need for such systems became more obvious. Most telemedicine systems are implemented on desktops or tablet devices [6,7]. However, these devices have practical limitations: (1) desktop systems have limited portability because they are installed in a fixed location; and (2) tablet device–based systems rely on manual input and control, which can hinder usability [9,10]. These issues could result in limited use of technology in real time, especially during complex care environments and time-critical patient scenarios because they demand the full cognitive attention and physical involvement of care providers [11].

**Figure 1:**
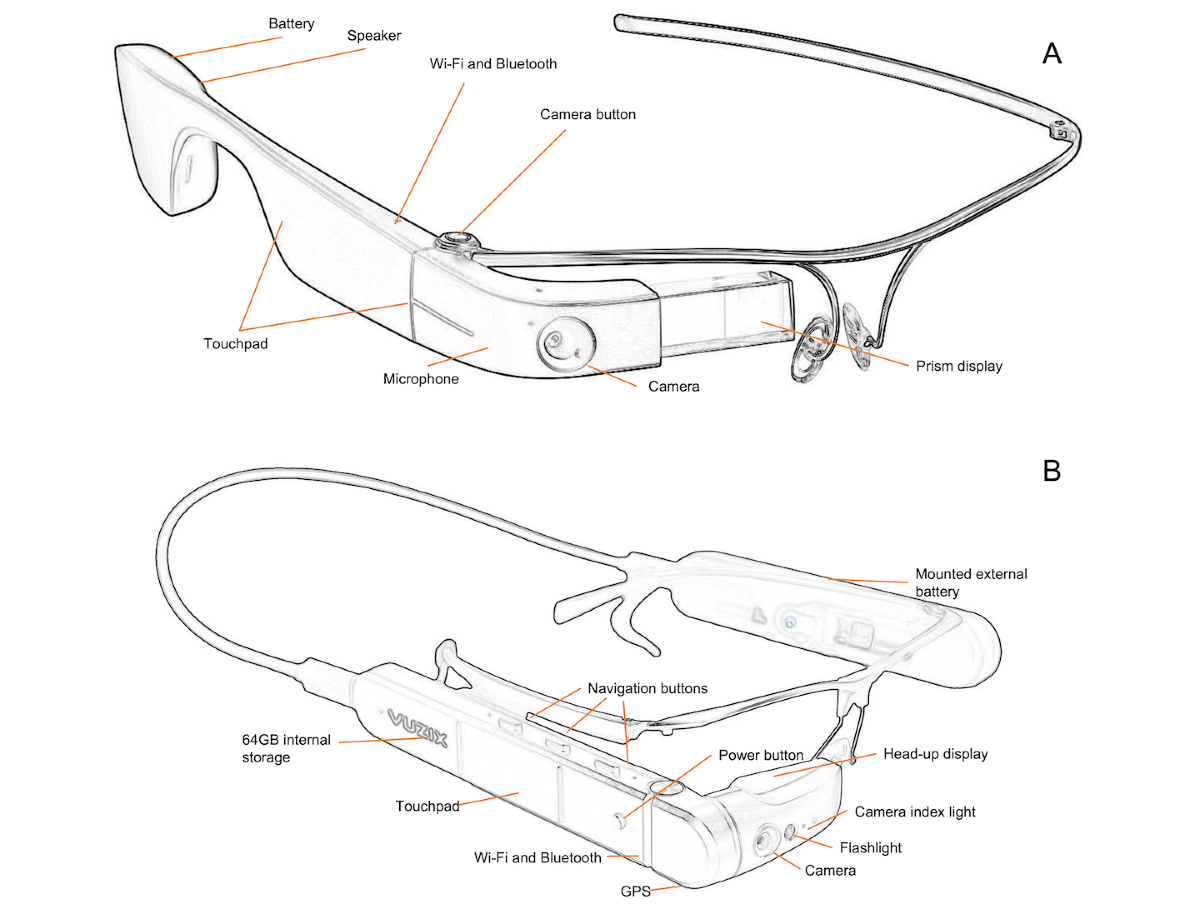
Examples of smart glasses with various hardware components labeled. (A) Google Glass. (B) Vuzix M400.

In recent years, the use of smart glasses—a computing device worn as a conventional pair of glasses (Figure 1)—has been gaining momentum in health care because they allow for real-time visual communication in a hands-free manner [12,13]. In particular, smart glasses can present both imagery and textual information within the wearer’s field of view (FOV) through a prism and enable videoconferencing for consults or second opinions via a front-facing camera. Since the introduction of smart glasses to the market, researchers have explored their applicability and usefulness in various medical settings and clinical scenarios [9], such as broadcasting surgeries to facilitate resident teaching [14], recording encounters with patients in wound care [15,16], assessing patients in mass casualty incidents [17], and supporting communication between prehospital and hospital providers [18,19].

### Objectives

As there is a growing interest in using smart glasses to support care coordination and communication across distributed care providers [9,11,20], the aim of this study was to synthesize the knowledge and experiences in this area, understand the benefits and limitations regarding adopting smart glasses as a telemedicine tool, and inform the design of future smart glass applications to better support remote care coordination. We focused on the use of smart glasses in care coordination in various clinical settings (eg, surgical operation, emergency care, and intensive care unit). Our specific research questions were as follows:

1. What are the general characteristics of prior research on using smart glasses for care coordination?
2. How was the system designed, used, integrated, and evaluated in supporting communication and care coordination across distributed care providers?
3. What types of challenges were identified by medical providers while they were using or testing the smart glass technology in practice?

These research questions were answered through a systematic literature review covering research within both health care and computer science fields.

Our work contributes the following to the medical informatics community: (1) an in-depth analysis and synthesis of prior research on the use of smart glasses for care coordination and communication; and (2) methodological and design implications for future research on smart glasses to improve distributed care coordination and communication.

## Methods

### Data Search

Our search started with discussing the search time frame and the most appropriate databases to use as well as search terms with experienced librarians. Using technology keywords such as “smart glasses” and “heads-up display,” along with health care keywords such as “distributed care” and “telemedicine,” a health librarian performed database searches for articles published between January 1, 2000, and March 1, 2022. We chose this time frame to capture the evolvement of this technology (ie, from early concepts such as head-worn displays [21] to smart glasses, which became a well-known concept after the introduction of Google Glass in 2013 [22]). The full list of search terms is presented in Textbox 1. We chose the following databases to cover research within both health care and computer science: ACM Digital Library, Cochrane Library, IEEE Xplore, Ovid MEDLINE, Embase, and Web of Science. A sample search strategy for Ovid MEDLINE is illustrated in Textbox 2. The database searches were set to include only studies published in peer-reviewed journals and conference proceedings in English. Literature reviews, dissertations, posters, and extended abstracts were excluded from the literature search. The retrieved citations were stored and managed using EndNote bibliographic management software (version X9; Clarivate).

**Keywords for literature search.**

**Search concepts and specific keywords**

- Smart glass: *smart glass, augmented reality glasses, heads-up display, head-mounted, head-worn, virtual reality, augmented reality, mixed reality, wearable technology, Google Glass, Vuzix, Epson Moverio*
- Clinical: *distributed care, remote care, telehealth, telemedicine, telecare, emergency care, pre-hospital*

**A sample search strategy for MEDLINE.**

**Search steps**

1. (“distributed healthcare” or “distributed care” or “remote care” or tele* or nursing or “long term care” or “home health” or “home care” or prehospital or pre-hospital or “emergency medical” or “emergency care” or paramedic* or ((clinical or surg*) adj3 (application* or use* or implementation*))).ti,ab,kf. or exp Telemedicine/ or exp Home Care Services/ or exp Emergency Medical Services/
2. ((smart adj1 glass*) or smartglass* or Hololens or picolinker or (google adj1 glass*) or vuzix or “epson moverio” or “augmented reality” or (AR and augmented) or “mixed reality” or “virtual reality” or (VR and virtual) or “wearable technology” or wearables or “heads up” or “head mounted” or “head worn”).ti,ab,kf. or wearable electronic devices/ or smart glasses/ or augmented reality/ or virtual reality/
3. Steps 1 and 2
4. Limit step 3 to (english language and yr=“2000-Current”)
5. (training* or education* or simulation* or telephon* or teleconferenc* or television*).ti. or exp *education/ or *telephone/ or *television/
6. Step 4 not step 5

### Article Screening and Selection

**Figure 2:**
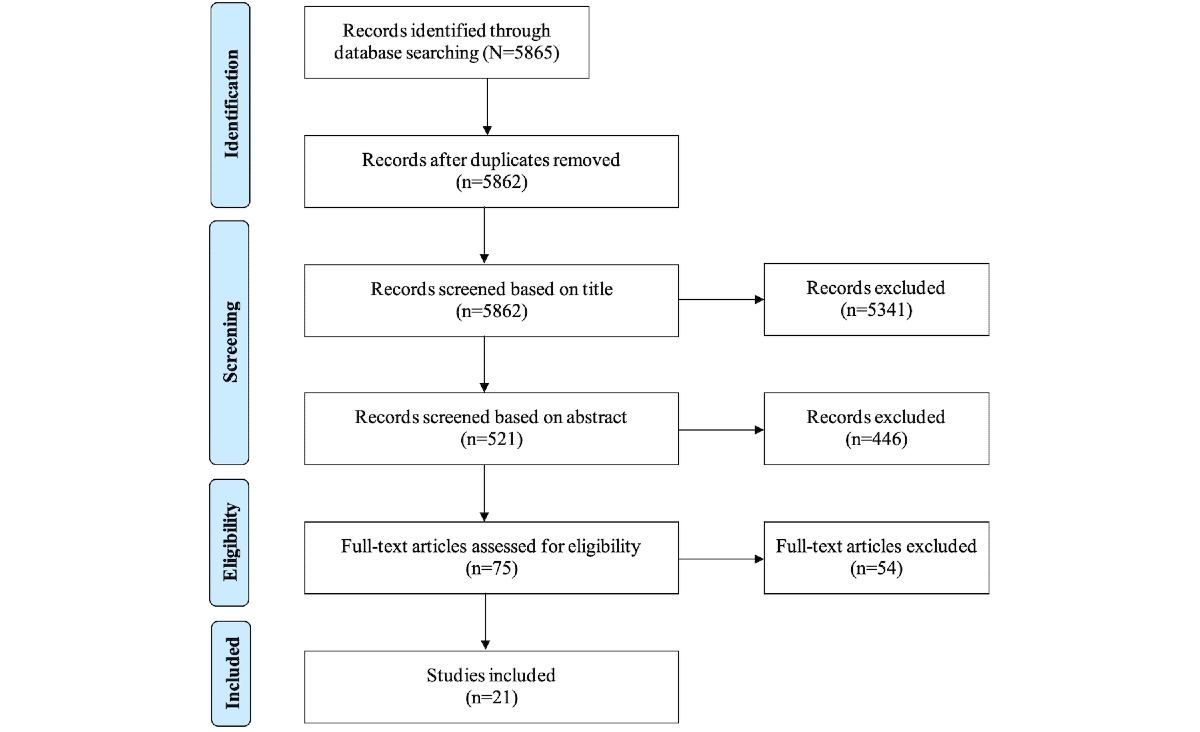
Information source and search strategy.

We used the PRISMA (Preferred Reporting Items for Systematic Reviews and Meta-Analyses) methodology to search and screen articles [23]. Figure 2 outlines the number of records that were identified, included, and excluded through different phases. More specifically, 5865 articles were identified through database searches, of which 5862 (99.95%) were included for screening after removing duplicates. Article titles were screened first, followed by abstract screening, to identify relevant articles. Of the 5862 articles, after screening of article titles, we excluded 5341 (91.11%); of the remaining 521 studies, 446 (85.6%), were excluded, leaving 75 (14.4%) for full-text review. After reviewing the full text of these 75 articles, we deemed 21 (28%) to be eligible for this systematic review.

Three authors (EB, KJ, and PG) independently screened all papers through the paper stack and selected relevant papers for inclusion. Two senior researchers (ZZ and MO) oversaw the whole article review and selection process. Any conflicts in selection decisions were resolved through discussion among all the authors during weekly group research meetings. The inclusion criteria were peer-reviewed articles that reported the use or testing of any smart glass technology and accompanying software in the context of communication and collaboration across distributed care providers. Articles were excluded if they only reported the use of smart glasses by an individual or in a collocated clinical setting or if they did not provide adequate supporting information, such as what clinical setting the smart glasses were used in and who used the technology.

### Data Extraction, Analysis, and Synthesis

Guided by the research questions of this study, 2 authors (KJ and EB) used a Microsoft Excel spreadsheet to extract, collate, and summarize data from the included studies, such as the country where the study was conducted, study objectives and scope, clinical scenarios, system evaluation methods, technology specifics, barriers and challenges, and a summary of study findings. Textbox 3 summarizes these data fields and their brief definitions. In addition to extracting the aforementioned metadata, we also assessed the technology readiness levels (TRLs) [24] of the systems tested in the reviewed studies. There are 9 different TRLs, ranging from level 1 (scientific knowledge generated underpinning hardware and software technology) to level 9 (actual system “flight proven” through successful mission operations). Two authors (KJ and EB) followed the metrics proposed in the study by Engel et al [25] and independently assessed TRLs for each system. They then compared and discussed their TRL evaluations until they reached agreement.

Two senior researchers (ZZ and MO) reviewed all the articles and analyses as a verification step. The research team met regularly to discuss the results. We performed the data analysis iteratively (ie, we went back and forth as more knowledge was obtained), as suggested by prior work [11,26]. A meta-analysis of the study results was not considered in this work owing to the heterogeneity of the study designs and results.

In the following section, we report information that was synthesized from the reviewed articles, including characteristics of the selected studies, system architecture and features, TRLs of the reviewed systems, system evaluation methods, and care providers’ perceived benefits and challenges of using and adopting smart glasses for distributed care coordination.

**Assessed article information and metadata.**

**Assessed information and brief definition**

- Study objectives and scope: the objective of the research and the purpose and scope of the use and test of smart glasses in each study (eg, patient care vs medical training)
- Clinical scenarios: the clinical domain and context in which the study was conducted
- Publication details: the type (eg, journal article vs conference paper), region, and year of the publication
- System infrastructure: the hardware, software, and network setup on both local and remote sites for establishing teleconsultation
- System features: the system features used, developed, or evaluated in each study
- System evaluation: the aspects of the smart glass system that were evaluated in the study and the methods used for system evaluation
- Benefits and challenges: the reported benefits and challenges of using smart glasses in improving communication and care coordination among distributed medical teams
- Major study findings: a summary of the major findings of a study

## Results

### General Characteristics of the Reviewed Studies

Of the 21 reviewed articles, 10 (48%) were conducted in the United States [18,19,27-34], and 2 (10%) were conducted for surgical teleproctoring between high-income countries and low- and middle-income countries (LMICs), such as between surgeons in the United States and Mozambique [35] and between experienced surgeons recruited from the United States and Germany and novice surgeons in Brazil and Paraguay [36]. The remaining studies (9/21, 43%) were conducted in different countries, such as Spain [37], China [38], Germany [39], France [40], Italy [41], Switzerland [42], Malaysia [43], South Korea [44], and Republic of the Congo [45]. The reviewed studies were conducted to assess the feasibility, effectiveness, and user experience of smart glasses in supporting remote patient evaluation and care procedure operation in a particular medical domain. The study objectives, along with major findings for each reviewed article, are presented in Multimedia Appendix 1 [18,19,27-45].

The clinical foci in these 21 papers vary: 9 (43%) focused on surgical settings [29,30,33-38,44], whereas 6 (29%) focused on the prehospital or emergency medical services domain [18,19,28,31,39,42]. The remaining studies (6/21, 29%) focused on intensive care [40,43], toxicology [27], ophthalmology [32], pediatric cardiology [41], and general medicine [45].

**Figure 3:**
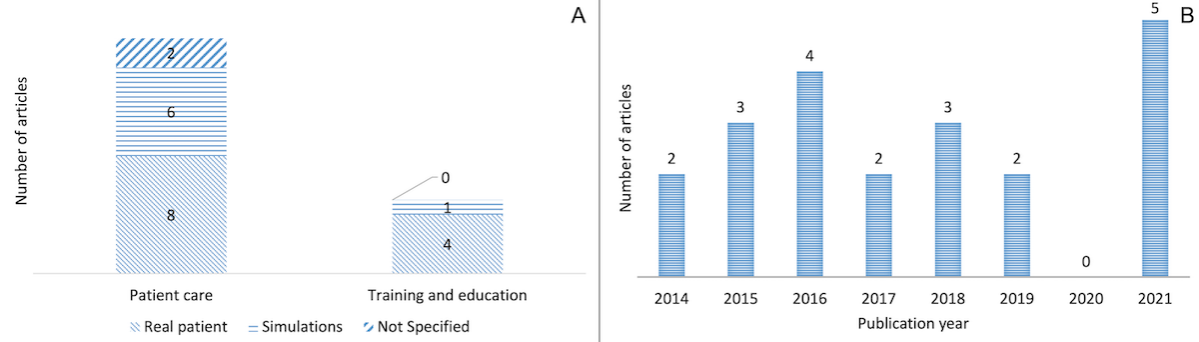
(A) The scope and testing environment of smart glasses in the reviewed articles. (B) The distribution of reviewed articles over the years.

The scope and purpose of the use of smart glasses among these studies vary. As shown in Figure 3A, the majority of the reviewed studies (16/21, 76%) used smart glasses to enable remote patient care and evaluation [18,19,27,28,30-32,37-45]. Of these 16 studies, 8 (50%) [27,28,30,32,37,38,43,45] tested smart glasses with real patients, 6 (38%) [18,19,31,39,40,44] conducted system testing in a simulated environment, and 2 (13%) [41,42] did not specify how the device was tested. The remaining studies (5/21, 24%) [29,33-36] leveraged smart glasses for training and teleproctoring purposes; of these 5 studies, 4 (80%) [29,34-36] tested the device with real patients, whereas 1 (20%) [33] tested the device in a simulated environment.

The reviewed articles were published between 2014 and 2021 (Figure 3B). It is noticeable that almost half of the reviewed articles (9/21, 43%) were published within the first 3 years of the release of Google Glass [22]. Subsequently, the number of studies on the use of smart glasses for supporting distributed care decreased until 2021. One possible explanation for this finding is that the use of smart glasses regained momentum right after the outbreak of the COVID-19 pandemic as researchers started exploring smart glass use to enable medical personnel to participate in remote assessment and consultation, with the aim of safeguarding patients and health care providers during the pandemic.

### System Architecture

**Figure 4:**
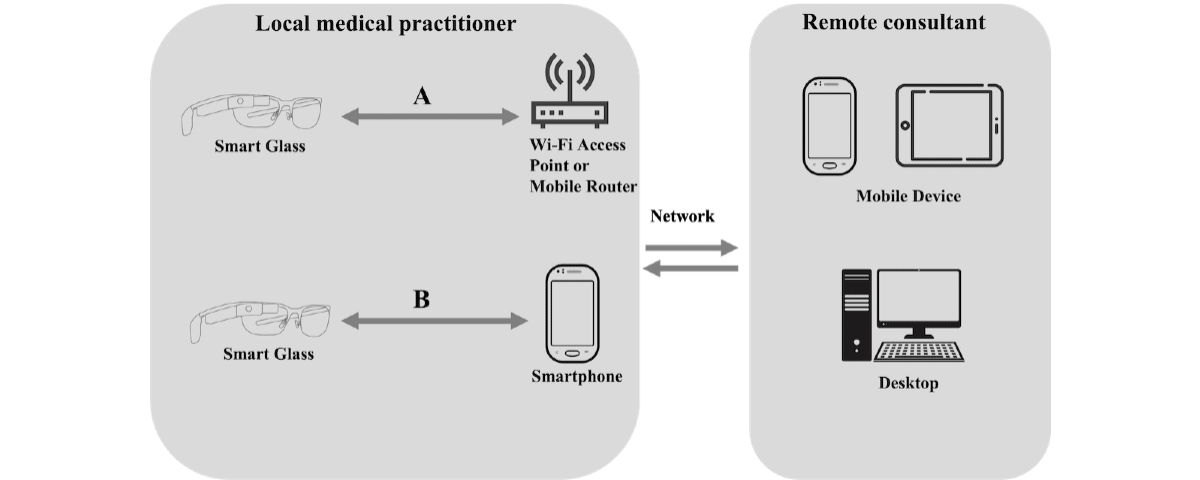
Common system architecture setups in the reviewed studies. (A) Smart glasses connected to a Wi-Fi network, a Wi-Fi hotspot, or a mobile router. (B) Smart glasses connected to a smartphone via Bluetooth or Wi-Fi.

Although the system architecture implemented in each study varied, there were some similarities across the reviewed studies. Typically, there are two types of technology setups on the local site: (1) smart glasses are connected to a Wi-Fi network, a Wi-Fi hotspot, or a mobile router to directly stream the first-person point-of-view to a remote consultant (Figure 4A); or (2) smart glasses are connected to a smartphone or a laptop via Bluetooth or Wi-Fi for video streaming and audio transmission (Figure 4B). The first approach was adopted by 52% (11/21) of the studies [19,27,28,31,32,36,38,39,41,43,44], and the second approach was used in 33% (7/21) of the studies [18,29,35,37,40,42,45]; for example, in the study by Diaka et al [45], the smart glasses were designed as an extension of a smartphone, which meant that the local wearer needed to initiate the call on the smartphone. Regardless of the system implementation method on the local site, the remote experts were usually equipped with either a computer or a mobile device (eg, a tablet device) to review and access the video stream and other multimedia data shared by the local medical practitioner (Figure 4). However, it is worth mentioning that in the study by Brewer et al [33], where smart glasses were used for surgical training, the remote expert (trainer) also wore a pair of smart glasses to view the video streamed from the learner.

**Figure 5:**
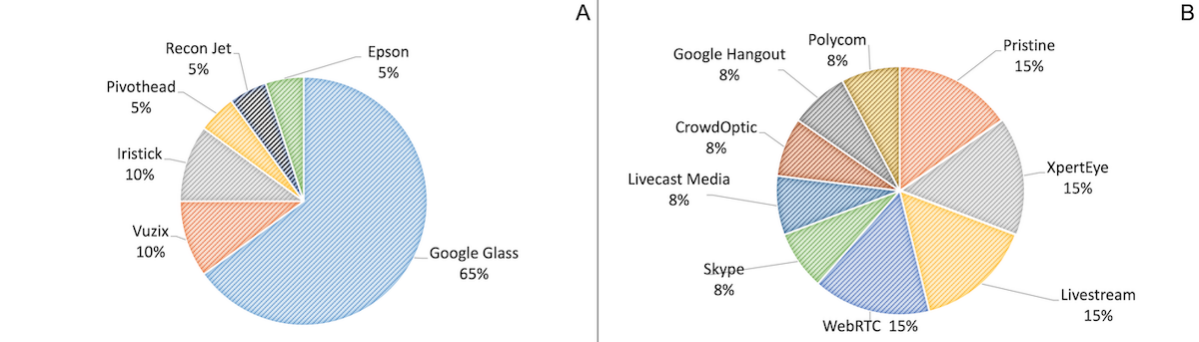
(A) Smart glass brands used in the reviewed articles. (B) Videoconferencing tools used in the reviewed articles.

As shown in Figure 5A, the reported brands of smart glasses in these studies included Google Glass [18,19,27-30,33-36,40,42,44], Vuzix [38,43], Iristick [37,45], Pivothead Original Series [32], Intel Recon Jet [31], and Epson Moverio BT-200 [41]. Google Glass was the most frequently used smart glass device (13/20, 65%). Another interesting observation is that all of the studies (21/21, 100%) used off-the-shelf, commercialized videoconferencing software (Figure 5B) such as Pristine Eyesight [19,27], AMA XpertEye [28,35], Livestream [18,36], WebRTC (enabled by Google) [42,44], Livecast Media [38], Skype [29], CrowdOptic [33], Google Hangout [34], and Polycom RealPresence Group 500 [32]. Most of the videoconferencing software used was compliant with the Health Insurance Portability and Accountability Act (HIPAA) rules, except in the case of the study by Cicero et al [18], where the researchers only tested the use of smart glasses in a simulated environment (real patient care was not involved).

### System Features

Although there was variation in the application scopes and domains, there were some common software features across the reviewed studies (Textbox 4). Real-time synchronous video and audio streaming from the local smart glass wearer to the remote consultant is the most common feature among the studies (19/21, 90%). In the case of the exceptions (2/21, 10%), because of technical limitations (eg, limited internet connection), the study by Gupta et al [30] first recorded patient care and evaluation using smart glasses and then transmitted the recordings to remote experts at a later time to simulate real-time telemedicine consults, whereas in the study by Hashimoto et al [34], researchers used Google Glass and an Apple iPhone to capture videos of a surgical operation and compared the video quality and its adequacy for safe use in telementoring.

Another noteworthy feature is enabling imagery and text-based remote guidance and annotation; for example, the remote consultant can annotate images captured from the live stream and project them back onto the local glass wearer’s visual field [35,37]. In 19% (4/21) of the studies [19,27,36,44], the remote consultant could use the texting feature to type messages that could be projected onto the smart glass display. These annotation features provide the remote consultant with more channels (in addition to audio and video) to direct and guide local medical practitioners to perform critical procedures.

Augmented reality (AR)—a technique that can enhance an individual’s visual experience of the real world through the integration of digital visual elements—was also tested in several studies. In Ponce et al [29], for example, AR enabled a remote surgeon to insert their hands or instruments virtually into the visual field of the local surgeon who wore smart glasses for real-time guidance, training, and assistance as needed. In another study [41], a remote specialist used AR-based markers to guide the execution of an echocardiographic examination performed by a local operator. The markers were overlaid on the ultrasound device and could be seen through the screen of the local operator’s smart glasses.

Other features of smart glasses reported in the studies included zooming in and out of the live stream video [35]; using voice commands [27,28,30,31] or head movements [27] to control, and interact with, the smart glass device; taking photographs [19,30,31,35]; automatically detecting the geographic location of on-site medical teams with the built-in GPS [31]; and presenting prehospital triage algorithm on the glass screen for decision support during mass casualty incidents [39].

**Summary of smart glass features as described in the reviewed studies.**

**System features**

- Real-time synchronous video and audio streaming [18,19,27-29,32-38,40-45]
- Record and forward video recordings [30]
- Imagery and text-based remote guidance and annotation [19,27,35-37,44]
- Augmented reality [29,41]
- Zooming in and out of the live stream video [35]
- Hands-free interaction with smart glasses [27,28,30,31]
- Taking photographs [19,30,31,35]
- GPS-based tracking of the geographic location of on-site medical teams [31]
- Presenting prehospital triage algorithm on the glass screen for decision support [39]

### TRLs of the Systems Tested in the Reviewed Studies

**Figure 6:**
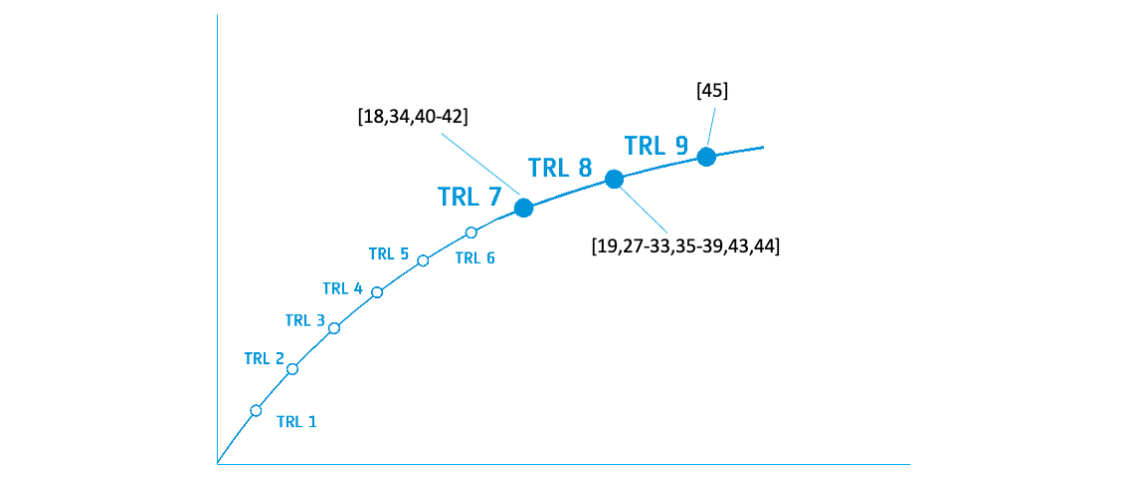
Diagram of technology readiness levels (TRLs) for the systems reported in the reviewed studies [18,19,27-45].

On the basis of our analysis, we found that the TRLs of all the systems used or tested in the reviewed studies ranged between 7 and 9. Our TRL assessment for each system is visualized in Figure 6 [18,19,27-45]. The reasoning for our assessment is summarized in Multimedia Appendix 2 [18,19,27-45].

The systems in 24% (5/21) of the studies [18,34,40-42] have a TRL of 7, which indicates that the technology is in the form of a high-fidelity prototype and has all key functionality available for demonstration and test; for example, in the study by Widmer and Müller [42], the Google Glass device on the local site was set up to connect with a computer application on the remote site for teleconsultation. This integrated system was only preliminarily tested by the research team but not in a simulated or real environment (a criterion for TRL 8); thus, its TRL was set to 7. It is worth mentioning that of these 5 studies, 3 (60%) [18,40,41] tested smart glasses in simulated scenarios; however, there were several reasons for their failure to meet the criteria for TRL 8, such as using non–HIPAA-compliant videoconferencing software, testing the technology with only 1 volunteer, or not fully integrating smart glasses with the network and remote devices.

The majority of the studies (15/21, 71%) [19,27-33,35-39,43,44] tested or used systems that met the criteria for TRL 8, indicating that they are actual systems in their final configuration and have been fully developed and tested in either simulated or real operational scenarios. However, these studies provided limited information regarding some criteria for TRL 9, such as whether the system had been fully integrated with other operational hardware and software systems (eg, database and hospital IT infrastructure), whether all system documentation had been completed, whether training on system use was available, and whether engineering support team was in place. Without such information, it is difficult to assess the readiness of these systems for large-scale deployment.

In comparison, only the system in the study by Diaka et al [45] was assessed to have a TRL of 9 because the system had been successfully operated on actual missions and tasks in the operational environment for a relatively long time (ie, more than a year). Furthermore, the system was fully integrated with other operational software, hardware, and network devices, as well as care delivery services (eg, moto-ambulances to facilitate patient referrals after teleconsultation).

### System Evaluations

#### Overview

The reviewed studies evaluated different dimensions of the smart glass system, including technical feasibility, effectiveness, and user experience. The details regarding the aspects of the smart glass system that were evaluated as well as the evaluation methods used in the reviewed studies are summarized in Table 1 and then elaborated on in the following sections.

**Table 1:** Summary of system evaluation details.

Evaluated dimensions	Specific evaluated aspects	Evaluation methods
Technical feasibility [27,34-36,44]	Success rate of established video teleconsultations between local and remote medical practitioners [27,36] Whether the quality of video and audio streaming was good enough for enabling video streaming [34-36,44]	Researchers’ observations of the successfulness of teleconsultations [27,36] Questionnaire [27,34-36,44]
Effectiveness [18,19,27,28,30-33,36,39,40,43]	Compared with in-person patient evaluation, whether the use of smart glasses could achieve similar performance and accuracy regarding patient evaluation and diagnosis [19,28,32,43] Compared with either mobile phone–based or no remote patient consultation, whether the use of smart glasses could lead to changes in clinical management and remote consultant’s confidence regarding diagnosis [18,27,30,39,40] Whether the use of smart glasses could improve medical training (eg, surgical operation) [33,36]	Comparison study between control (without smart glass support) and treatment (with smart glass support) groups [18,19,28,32,39,40,43] Questionnaire [33] Exit interview [36]
User experience [18,19,27,29-31,35,37-41,43-45]	Usability of smart glasses [19] Opinions regarding using and adopting smart glasses in practice [18,27,30,31,35,37-41,43-45]	Survey [18,19,27,29-31,35,38-40,43,44] Interviews and observations [18,31,35,45]

#### Technical Feasibility

Several studies assessed whether the smart glass technology was a practical means to support care coordination and communication in different contexts, such as teletoxicology consults [27] and remote surgical teleproctoring [34-36,44]. The main measurements included the success rate of established video teleconsultations between local and remote medical practitioners and whether the quality of video streaming was acceptable and good enough to allow for real-time, seamless guidance and assistance. The technical feasibility was primarily determined by the researchers’ observations and the users’ ratings via questionnaire; for example, in a study evaluating the feasibility and acceptability of Google Glass for teletoxicology consults [27], questionnaires were administered immediately after the study to elicit remote consultants’ opinions regarding whether consults through smart glasses were considered successful and the technical feasibility of using smart glasses for teleconsultation.

#### Effectiveness

Of the 21 reviewed studies, 10 (48%) evaluated the effectiveness of smart glasses, that is, whether this novel technology could improve patient care and decision-making compared with current approaches (eg, no remote consultation, in-person patient evaluation, or consultation via telephone) [18,19,27,28,30,32,33,39,40,43]; for example, in some settings where remote consultations were usually accomplished via telephone or radio, which typically do not support visual communications [27,30], researchers compared using such traditional communication mechanisms with using smart glasses to determine whether the use of smart glasses could lead to changes in clinical management and the remote consultant’s confidence regarding diagnosis.

Of these 10 studies, 7 (70%) [18,19,28,32,39,40,43] conducted an experiment with a control group (no smart glasses and either in-person consultation or no remote consultation at all) and an intervention group (with smart glasses) to measure whether using smart glasses could increase the quality and accuracy of patient diagnosis while reducing the time needed to perform patient care; for example, in the scenario of patient triage during mass casualty incidents [19], researchers asked 2 emergency medicine (EM) physicians (control group) to make triage decisions after examining the simulated patients in person as 2 other EM physicians (intervention group) simultaneously evaluated the same group of patients via real-time point-of-view video stream from a paramedic wearing Google Glass. They then used the agreement within and among the groups of EM physicians on the need for immediate trauma evaluation to determine the effectiveness of smart glasses for supporting patient triage.

#### User Experience

Of the 21 studies, 15 (71%) examined end users’ experience and perceptions to some extent with regard to using smart glasses in their work [18,19,27,29-31,35,37-41,43-45]. The primary methodology used for eliciting user experience was a survey, which was adopted by 80% (12/15) of these studies [18,19,27,29-31,35,38-40,43,44]; for example, in a recent study [43], a survey was sent to the participants on completion of the study to assess acceptance, satisfaction, overall impact, efficacy, and potential of adopting smart glasses as an alternative method of teleconsultation in neurosurgery. Among these 12 studies that administered a survey, 9 (75%) specifically reported the number of participants, which ranged between 2 and 276. Other methods such as interviews and observations were also used to gather more qualitative, in-depth insights from end users [18,31,35,45]. In particular, of these 4 studies, 2 (50%) [31,35] conducted interviews in conjunction with a survey.

It is also worth mentioning that of the 15 studies, 2 (13%) [19,31] specifically focused on evaluating the usability of smart glasses, that is, whether smart glass technology is perceived as easily usable by, and acceptable to, medical professionals. Another study [30] also examined patient perceptions of medical providers wearing smart glasses with recording capability. Finally, of the 15 studies, 5 (33%) [29,37,38,41,42] mentioned that they collected end users’ opinions and experiences but did not specify the methods they used.

### Benefits and Challenges of Using and Adopting Smart Glasses for Teleconsultation

#### Benefits

Our reviewed work highlights the advantages of smart glasses in improving communication and care coordination among distributed medical teams because this technology enables local medical providers to share visual information and perform teleconsultation in a hands-free manner. Regarding the effects on clinical care and patient outcome, the studies reported that smart glasses could shape clinical management and boost remote consultants’ confidence in clinical care [27,30], achieve diagnostic accuracy comparable with that achieved in in-person patient examination [19,28,32,43], improve proficiency and performance of the clinical tasks [31,33,35,38-40], and lower the medical service cost and improve quality of life for people in rural areas or LMICs [36,38]. Finally, many studies reported positive user perceptions, acceptance, and satisfaction with the use of smart glasses [19,27,29-31,35,38,39,41,43,45].

Notwithstanding these reported benefits, the reviewed studies also highlight a set of challenges and user concerns regarding the adoption of smart glasses in practice. We grouped them into 4 main categories: technical challenges, human factors and ergonomics, privacy and security concerns, and organizational challenges (Textbox 5).

**Challenges to using and adopting smart glasses in practice.**

**Technical challenges**

- Unstable or low-bandwidth internet connections [18,19,29,33,35,36,39,44]
- Battery drain becomes higher during video streaming [18,29,39]
- The microphone is unable to filter out background noise [18,29]
- Screen contrast and readability issues in bright or dark environments [18]
- Image distortion owing to overexposure to room light [18,29,35]
- Smart glass see-through screen is too small for easy interaction [41]
- Difficulty controlling video streaming software [18,35,38]
- Lack of a lock function to prevent the possibility of inadvertently halting the video streaming and ability to opt out of frequent software updates [18]

**Human factors and ergonomics**

- Compatibility issues with wearer’s glasses or personal protective equipment [27,29,35,37,39-41]
- Misalignment between the direction of gaze and range of smart glass camera [29,35,37,40,41,43]
- Voice control function could be problematic [18,30]
- Added distractions for medical professionals [31]

**Privacy and security concerns**

- Concerns regarding violations of patient privacy and data breach [28-30,43]

**Organizational challenges**

- Added workload for medical professionals [39]
- Costly device and software [35]
- End users have limited experience with, and prior knowledge of, smart glasses; need extensive equipment and software training [27,37,41,43]

#### Technical Challenges

The reviewed studies reported a variety of technical challenges that may impede the effective use of smart glasses in teleconsultation. These challenges are mainly related to internet connections, hardware limitations, and software reliability. More specifically, because smart glasses require a high-speed network to transmit visual media (eg, video streaming, audio, and pictures), unstable or low-bandwidth internet connections were seen as a major technical barrier because this issue would compromise video and audio quality, leading to breakdowns in communication and loss of patient information [18,19,29,33,35,36,39,44]. This is more evident in low-resource or out-of-hospital settings where medical practitioners have limited access to the internet; for example, because Wi-Fi is not steadily available in the prehospital environment, the problem with internet connections was commonly reported in this domain [18,19,39]. One practical and successful solution used by a study in prehospital communication [31] was using a mobile router to provide a fault-tolerant network that ran independent of Wi-Fi and other external networks, allowing for deployment at any location.

Regarding hardware limitations, medical professionals were concerned about battery life (eg, the battery could get drained quickly during video streaming) [18,29,39], microphone sensibility (eg, not being able to filter out background noise) [18,29], screen contrast and readability (eg, hard to read the screen in extremely bright or dark environment) [18], image quality (eg, the image could be distorted because of overexposure to room light) [18,29,35], and small screen for interaction [33,41,44].

Issues regarding software were primarily related to controlling and interacting with the video streaming software; for example, 14% (3/21) of the studies [18,35,38] mentioned difficulties regarding zooming in or out during video streaming; as such, the smart glass wearer needs to bring their face close to the patient. Other software issues included the lack of a lock function to prevent the possibility of inadvertently halting the video streaming and the inability to opt out of frequent software updates [18].

#### Human Factors and Ergonomics

Many issues related to the interactions between users and the smart glass system were also reported. First, 38% (8/21) of the studies [27,29,33,35,37,39-41] highlighted the compatibility issue with users’ spectacles or personal protective equipment. In particular, fitting the smart glass headset onto surgical loupes was problematic, interfering with the surgeon’s ability to wear such devices [35]. Some users had to remove their spectacles to wear the smart glass headset or tie up their hair to prevent the glass camera from being hidden [40]. Second, the difference in line of sight—misalignment between what the glass wearer sees (eg, the direction of gaze) and what the camera captures (eg, range and angle of the camera)—was also cited as a major barrier [29,35,37,40,41,43]. This issue was often attributed to the limited FOV of smart glasses [33,44]. This misalignment problem could be worsened owing to sudden head movements and frequent relocation of the smart glass wearer or the patient’s unpredictable movements because these could cause motion blur for remote experts or consultants and make it difficult for them to identify the clinical situation [44]. Third, although the reviewed studies reported that their participants perceived that the smart glass was easy to use overall, usability issues still exist; for example, the voice control function did not work perfectly and thus required the user to remove their gloves to use the built-in touchpad or buttons to operate the device, such as starting or stopping the video call [18,30]. In another study, smart glasses were reported to be a distraction for medical practitioners [31].

#### Privacy and Security Concerns

Patient privacy and data security issues were perceived as important to address because smart glasses can transfer or even store sensitive patient data [28-30,43]. These studies stated that any implementation of smart glasses must not only comply with HIPAA requirements but also alleviate patient concerns about any potential privacy violation or misuse of their data [30,43].

#### Organizational Challenges

As medical professionals have limited prior knowledge of using the novel smart glass technology (compared with their experience of using smartphones or tablet devices), a few studies mentioned that user training is necessary to increase efficiency and reduce human errors in system operation [27,33,37,41,43]. In addition, the smart glass technology is costly; for example, as McCullough et al [35] reported, the cost of a yearly contract for a piece of wearable hardware and the videoconferencing platform is approximately US $7000. Such high costs could become a critical barrier to adopting this technology at scale, especially for those health care providers who have limited resources. Finally, integrating smart glasses into the current workflow is a prominent challenge; for example, Follmann et al [39] reported that adopting smart glasses in prehospital triage and communication added more workload to emergency care providers in the field and took markedly more time compared with not using smart glasses.

## Discussion

### Methodological Implications

In this work, we conducted a systematic review of studies focused on the use and application of smart glasses in supporting care coordination and communication among distributed medical teams. Of the 5862 papers included for screening, only 21 (0.36%) met our criteria, highlighting the paucity of studies examining the feasibility, effectiveness, and user experience of using smart glasses as a telemedicine tool. Furthermore, the studies were mostly conducted in the United States and a few other high-income countries (eg, Italy, Germany, and France). One possible explanation is that smart glass technology is costly, hindering its adoption in LMICs and low-resource settings. However, 14% (3/21) of the reviewed studies [35,36,45] revealed the substantial benefits that smart glasses could bring to LMICs and rural areas, such as providing remote training and mentoring and more accurate instructions to the field medical practitioners in low-resource settings who otherwise have limited access to remote experts. Given such benefits, more future work is needed to expand the research of smart glasses to LMICs.

Another interesting observation is that all the reviewed studies (21/21, 100%) only used off-the-shelf hardware and software without involving users in the system design process. Prior work has suggested that it is critical to involve users and understand user requirements in the early phase of system development to identify and address potential usability and technical issues [6,46,47]. In addition, regarding the methodology for eliciting user opinions, out of 15 studies conducted user evaluation, 33% (5/15) of them did not specify what questions they asked, how the questionnaire was developed, and what procedure was followed. Despite the user-friendliness of health care information technology being a determinant factor for user adoption and acceptance [48,49], the usability of smart glasses was neglected by most of the studies (19/21, 90%), with only the studies by Broach et al [19] and Demir et al [31] specifically examining this aspect. These facts highlight the need to adopt a *user-centered design* approach in the development of smart glass technology by placing users at the center of the system design process from inception to implementation and deployment.

A similar concern is that a few of the reviewed studies (4/21, 19%) only recruited a small number of study participants (eg, 2 health care professionals) to participate in their user studies (eg, survey or interview). In addition, some of the studies (5/21, 24%) did not report the details of their user research, including the number of participants. These findings may suggest that the important role of user research was not recognized in some of the reviewed studies (9/21, 43%), and their results might not be generalizable because of the limited number of study participants. Given these study limitations, we argue that involving human-computer interaction researchers in such type of research and establishing close collaborations between these researchers and health care domain experts are critical and much needed, as demonstrated in the study by Schlosser et al [50].

Finally, almost all of the reviewed studies (20/21, 95%) focused on evaluating the smart glass technology either from a technical perspective or a clinical perspective, while neglecting other important factors that could substantially affect the use and adoption of this technology, such as workflow, teamwork, policies, and organizational cultures. As prior work has argued [51], an ongoing challenge to the successful implementation and deployment of health IT (HIT) interventions is to operationalize their use within the workflow of a complex health care system; for example, a new technology could disrupt current clinical work, causing not only frustrations for medical providers but also patient safety issues [52-54]. When this problem occurs, not surprisingly, medical practitioners are left with no choice but to bypass the technology or adopt informal, low-tech, potentially unsafe workarounds that deviate from the formal protocol [55,56]. As such, researchers have highlighted the importance of examining the design, use, and application of HIT interventions through the lens of a sociotechnical perspective [55-57]. This approach allows researchers and practitioners to understand the complex interrelations between various social and technical elements of systems that are equally important in determining the success of HIT adoption in a health care organization. In line with this argument, we believe that more research adopting a sociotechnical model [51,58] is needed to investigate the factors (eg, human-computer interaction, workflow and communication, internal organizational features, and external rules) that contribute to the uptake of smart glasses in routine use.

### Design Implications

The reviewed studies revealed a set of challenges and barriers to adopting and using smart glasses in practice; for example, a commonly cited technical challenge is internet connection quality—smart glasses rely on a high-bandwidth internet network for streaming videos and transmitting other visual media data (eg, high-resolution pictures, texts, and augmented objects). However, this technical requirement could be challenging to fulfill, especially in low-resource or out-of-hospital settings [59]. With the rapid development of 5G technology, this technical barrier might be overcome in the near future; for example, a study [60] showed that 5G technology could not only enable safe and efficient complex surgical procedures during telementored surgery but also lead to a very high degree of surgical team satisfaction. In addition to internet connections, other technical improvements suggested by the reviewed studies include increasing the memory space of smart glasses to store more information, adding autofocus and stabilization features to the smart glass camera, and improving the camera resolution [35].

Human factors and usability issues make up another set of important considerations for smart glass designers and developers; for example, the difference in line of sight between the local medical practitioner and remote consultant impeded the remote consultant from seeing exactly what the smart glass wearer’s eyes were fixed on. In addition, the limited FOV further complicated the video transmission to the remote experts. One reviewed study [44] experimented by attaching a mirror to the smart glass to increase the FOV of the local practitioner by transmitting both the wearer’s front view and their hand operations below the camera to the remote experts. However, the video received on the other end by the experts was deemed confusing. Another viable solution suggested by prior work [59] is using more advanced mounting techniques to make sure that the smart glass can sit steadily on the wearer’s head to align their visual field with the camera range. Another interesting issue brought out by a few of the studies (5/21, 24%) was the necessity of enhancing user interactions with the smart glass, such as offering more hands-free interaction mechanisms (eg, using head movements to control the device) [35] and enabling the user to zoom in and out during video streaming as well as pan the image [38].

Current smart glass applications are stand-alone and limit their potential. The data collected and transferred through smart glasses can best benefit patient care tasks if they can be incorporated into, and fully integrated with, other HITs such as electronic health records or clinical decision support systems. Interoperability issues (eg, standardized terminology) should be considered when deploying and integrating smart glasses into complex health care systems.

Other important design considerations that need full attention for developing and deploying the smart glass technology include (1) ensuring that the software is compliant with HIPAA requirements to protect patient privacy and data security, (2) integrating smart glasses into the workflow to minimize the disruption to medical practitioners’ work, and (3) providing sufficient training to end users.

### Study Limitations

Defining the search keywords was difficult. To generate a comprehensive and relevant list of keywords, we iteratively discussed and selected the keywords for the search based on suggestions from the health librarian and a review of systematic review articles regarding smart glasses. Another limitation is that we did not assess the quality or impact of the results from the included articles. A meta-analysis was not feasible because of the heterogeneity of the study designs and results.

### Conclusions

Smart glasses were found to be an acceptable and feasible tool in enabling visual communication and information sharing among distributed medical teams. Despite the high potential of this novel technology, the reviewed articles pointed out a set of challenges that need to be addressed before the wide deployment of this technology in complex health care systems. Thoughtful system design involving end users from the beginning and improved hardware and software reliability are needed to improve the usefulness and usability of smart glasses for medical practitioners [11,59]. We suggest that more user-centered design and evaluation research is needed to examine and evaluate medical professionals’ needs and perceptions and determine how to design smart glass technology to meet their needs. In addition, more research is required to elucidate how smart glasses affect the workflow of medical professionals in complex care environments.

## Appendix

Multimedia Appendix 1 Summary of study objectives and major findings.

Multimedia Appendix 2 Technology readiness levels of the systems reported in the reviewed studies.

## Abbreviations

AR: augmented reality
EM: emergency medicine
FOV: field of view
HIPAA: Health Insurance Portability and Accountability Act
HIT: health IT
LMIC: low- and middle-income country
PRISMA: Preferred Reporting Items for Systematic Reviews and Meta-Analyses
TRL: technology readiness level
