# Supplementary material for: Smart Glasses for Supporting Distributed Care Work: Systematic Review
Source: JMIR Med Inform. 2023 Feb 28;11:e44161. doi: 10.2196/44161 (PMC10015357; doi:10.2196/44161)
Supplement: Multimedia Appendix 1 [file medinform_v11i1e44161_app1.docx]

**Table**: Summary of study objectives and major findings

| **Authors** | **Study Objective** | **Major Findings** |
| --- | --- | --- |
| Broach et al. [19] | To evaluate the usability and reliability of smart glasses for secondary triage during mass casualty incidents. | - Most first responders positively perceived the use of smart glasses for augmented triage and decision support and felt that the smart glass technology was easy to use and would not be a major impediment to their work in the field. - Using a smart glass platform to perform secondary triage remotely prior to patient arrival in the emergency department (ED) would have similar reliability compared to in-person triage upon patient arrival in ED. |
| Chai et al. [27] | To examine the feasibility and acceptability of using Google Glass to assess the poisoned patient by remote toxicology consultants. | - Consults through Google Glass were considered successful by remote consultants in 89% of cases. - The confidence of remote consultants in diagnosing a specific toxidrome increased to 94% after a virtual exam compared to phone consultation. - The virtual exam through Google Glass changed the management of the patient in 56% of cases. |
| Noorian et al. [28] | To assess the feasibility and inter-rater reliability of using smart glasses in the remote evaluation of stroke-related neurologic deficits in patients with suspected acute stroke. | - The smart glass interface was well designed for use in the field and performed comparably to bulkier hospital systems. - Achieved excellent inter-rater reliability in assessment of neurologic deficit on the National Institutes of Health Stroke Scale (NIHSS) between a remote examiner accessing the patient via smart glasses and an in-person examiner. |
| Cicero et al. [18] | To determine the feasibility of using Google Glass in disaster triage, and its effect on the accuracy of triage or the time needed to perform triage. | - Using smart glasses to evaluate disaster victims in the field did not significantly increase triage accuracy but required more time than conventional triage. - Of all the mis-triaged patients, those in the intervention group (consultation with a remote physician via smart glasses) tended to be under triaged, while almost 30% of mis-triage instances for the control group (no smart glass) were over triaged. |
| McCullough et al. [35] | To demonstrate the feasibility of using Google Glass to expand access to training opportunities in reconstructive surgery in low-resource settings. | - Both the mentor surgeon and field surgeon reported that the smart glass technology was very helpful for surgical training. - Technical limitations, such as image distortion, video-stream latency, and connection disruption, were experienced by surgeons. |
| Ye et al. [38] | To assess the feasibility of using 4G technology-enabled smart glasses or smartphones for wound management. | - Multiway real-time communication occurred without any difficulty and the resolution of the video was acceptable. - Both local surgeons and patients showed good acceptance of this wound care model and the used technologies. |
| Drummond et al. [40] | To determine whether real-time video communication between the first responder and a remote intensivist via Google Glass improves the management of in-hospital pediatric cardiopulmonary arrest (pCPA) before the arrival of the ICU team. | - Local first responders found using smart glass to consult with an intensivist helpful. - Using smart glasses did not decrease no-blow and no-flow fractions during the first five minutes of a simulated pCPA, but improved the quality of the insufflations and chest compressions provided. - The pCPA was more frequently interrupted by discussions with the intensivist in the smart glass group than in the control group. |
| **Authors** | **Study Objective** | **Major Findings** |
| Gupta et al. [30] | To explore the use and potential value of asynchronous, near real-time communication via Google Glass which avoids transmission issues associated with real-time video streaming to facilitate remote surgical consults in the emergency department. | - Surgical consultants’ confidence in their management plan increased in 44% of cases after they reviewed the video recorded by smart glasses. - The wearers (physicians) noted that the smart glass did not generally interrupt their workflow during the physical examination or patient interview. - A survey with 276 patients revealed that the majority of patients are amenable to the addition of wearable technology with video functionality to their care. |
| Follmann et al. [39] | To evaluate the technical feasibility and effectiveness of smart glasses for triage support during a mass casualty incident. | - The accuracy of the triages was markedly increased with smart glass support, but triages with smart glasses took longer than conventional triage. - 73% of participants reported a good level of acceptance, and good to very good usability of smart glasses. |
| Ponce et al. [29] | To report the experience with using real-time augmented reality (AR) and wearable computing devices such as Google Glass in surgical operations. | - Google Glass was more convenient and less obtrusive than a camera mounted in the operating room. - Both local surgeons and remote consultants reported a positive experience. - Using AR technology (e.g., inserting remote surgeon's hands into the local surgeon's surgical field) allows for more precise instruction than through verbal interaction. |
| Demir et al. [31] | To report the design, development, and evaluation of an integrated system for multiple casualty management and triage. | - The system includes smart glasses (with augmented reality technique) and portable network devices for first responders and paramedics, and an intelligent dashboard with map view and digital notes features for medical incident commanders. - The system was iteratively designed and evaluated, using methods such as heuristic evaluation, surveys, and interviews, as well as usability evaluation. |
| Widmer and Müller [42] | To describe an application using Google Glass to allow paramedics to access information from a hospital system and send information from an accident site. | - The application was able to take a photo and send it to a medical image retrieval system along with keywords in order to retrieve similar cases. - Preliminary testing revealed that this application could potentially help the decision-making task in the pre-hospital domain. |
| Rio et al. [41] | To evaluate the impact of augmented reality (AR) enabled by smart glasses in remotely assisted ultrasound exams. | - Overall impressions about the use of AR and smart glasses were positive. - The system was evaluated as a valid tool for improving communication and helping specialists when remotely guiding a non-expert operator. |
| Diaka et al. [45] | To demonstrate the effectiveness of using smart glasses to improve primary healthcare services, especially referrals to the district hospital in the rural area of the Democratic Republic of the Congo. | - The total number of consultations increased significantly in the intervention health centers. - The number of referrals to the hospital remained stable, but an increased proportion effectively arrived in the hospital. - All stakeholders reported being very positive about the technology. - Benefits of introducing smart glasses to low-resource settings include but are not limited to improved medical skills of health center staff, improved referral system, improved convenience and access to care for the underserved population. |
| **Authors** | **Study Objective** | **Major Findings** |
| Ho et al. [32] | To evaluate the use of smart glasses with video recording capability in streamed strabismus examination. | - The diagnostic accuracy of real-time telemedicine via smart glasses was comparable to in-person examination. - Image quality was not a significant barrier to rendering diagnoses. |
| Martínez-Galdámez et al. [37] | To highlight the potential benefits and limitations of using smart glasses in Neurointerventional procedures. | - One potential advantage of the smart glass is that the visual field of the operation site is not disturbed by external elements or the operator’s head. - Another advantage is that smart glasses don’t need a complex installation, making them more accessible and replicable. - Challenges in using smart glasses in this environment included difficulty to conform the operating space to the fixed view of the camera of smart glasses, and the video streaming could be disturbed by the patient's unpredictable movements. |
| Munusamy et al. [43] | To determine if telemedicine delivered through smart glasses was a feasible and effective alternative method for conducting ward round on neurocritical care patients during the COVID-19 pandemic. | - This alternative approach received a high user acceptance and satisfaction rate. - Virtual ward rounds using telemedicine via smart glasses on neurosurgical patients in critical care were feasible, effective, and widely accepted as an alternative to physical ward rounds during the COVID-19 pandemic. |
| Brewer et al. [33] | To test whether the introduction of a smart glass system allowing the remote instructor to view otherwise “blind” areas of trainee in the operative field could improve the training of surgeons. | - Introduction of the smart glass system did not improve the time to task completion. - The accuracy of needle placement was significantly improved in the condition of using smart glasses. - Most of the participants (surgical trainer and trainee) deemed the device unobtrusive, easy to use, and useful for communication and instruction. |
| Hashimoto et al. [34] | To assess the safety of using Google Glass by measuring the video quality of a telementoring sessions. | - A total of 50% of invited surgical attendings rated the Google Glass video as fair with the other 50% rating it as bad to poor. - The video quality of Google Glass was deemed inadequate for telementoring in open surgery. |
| Datta et al. [36] | To evaluate the feasibility of using smart glasses and web-based communication for long-term proctoring in an international setting. | - The smart glasses and web-based communication tools made collaborative surgical education efforts between international experts and local care providers in resource-poor regions feasible and effective. |
| Yoon et al. [44] | To determine the usefulness, usability, and feasibility of using latest Google Glass (Glass EE2) and a desktop user interface to establish real-time video and audio connection in emergency care settings. | - The latest Google Glass demonstrated sufficient technical performance with a satisfactory level of image quality and auditory communication. - However, it is still not easy to identify clinical situations, patients’ conditions, and trainees’ performance. - Barriers in using the system are related to network and connectivity, narrow field of view and motion blur in videos captured by Google Glass. |
