# Supplementary material for: Smart Glasses for Supporting Distributed Care Work: Systematic Review
Source: JMIR Med Inform. 2023 Feb 28;11:e44161. doi: 10.2196/44161 (PMC10015357; doi:10.2196/44161)
Supplement: Multimedia Appendix 2 [file medinform_v11i1e44161_app2.docx]

**Table**: Technology readiness levels of the systems reported in the reviewed studies

| **TRL** | **Study** | **Reasoning/Justification** |
| --- | --- | --- |
| TRL 7 (n=5) | Widmer and Müller [42] | Google Glass was set up to use on the local site to capture audio and video data, which were then transmitted to an android smartphone over a Wi-Fi network. The smartphone application sent the video and audio to the hospital. The device used on the remote site (a computer application) was set up for teleconsultation. Videostreaming was enabled by WebRTC. The smart glass system was preliminarily tested by the research team. The details of the testing environment was not provided. |
|  | Hashimoto et al. [34] | The study aimed to assess the video quality of Google Glass for telementoring in open surgery. 34 surgeons blindly compared video captured with Google Glass versus an Apple iPhone 5 during a surgery. No system infrastructure or internet was set up. |
|  | Cicero et al. [18] | The smart glass system used on the local site (Google Glass) was a commercial product which had been fully developed and tested by the manufacture. However, the videostreaming software was not compliant with HIPAA requirements. The device used on the remote site (a computer) was functional enough for viewing video streaming of the simulated patients. Both systems were fully integrated and tested in simulated scenarios. |
|  | Drummond et al. [40] | The smart glass system used on the local site (Google Glass) was commercial products which had been fully developed and tested by its manufacture. This study used a personal laptop located in another room to simulate a remote site. Videostreaming was established by a HIPAA compliant commercial product (AMA). However, the smart glass was connected with the personal laptop using a 20-meter USB cable instead of wireless internet. This system configuration is not adaptable to long-distance teleconsultation. The entire system was used and tested in simulated scenarios. |
|  | Rio et al. [41] | The smart glass system used on the local site (Epson) was a commercial product which had been fully developed and tested by its manufacture. An augmented reality system was developed specifically for the remote experts to use for telementoring. The system was only preliminarily tested on one volunteer. In addition, the details of this preliminary test were not provided. |
| TRL 8 (n=15) | Broach et al. [19] | The smart glass system used on the local site (Google Glass) was a commercial product which had been fully developed and tested by its manufacture. The system used on the remote site (a desktop application running a secure portal on a web browser) was fully functional. Both systems were fully integrated. Videostreaming was established by a HIPAA compliant commercial product (Pristine). The integrated system was used and tested in simulated scenarios. |
|  | Ye et al. [38] | The smart glass system (Vuzix M100) used on the local site was a commercial product which had been fully developed and tested by the manufacture. The system used on the remote site (a web portal) was also functional. Both systems were fully integrated. Videostreaming was established by a commercial product (Livecast). The users showed good acceptance of the technology. The integrated system was tested in real settings. However, the study was implemented in a short time and the number of subjects was small (n = 5). In addition, limited information was provided regarding some criteria of TRL 9, such as whether the system had been fully integrated with other operational hardware/software systems (e.g., database, hospital IT infrastructure), whether all system documentation had been completed, and whether sustaining engineering support team was in place. |
| **TRL** | **Study** | **Reasoning/Justification** |
| TRL 8 (n=15) | Follmann et al. [39] | The smart glass system used on the local site was a commercial product which had been fully developed and tested by its manufacture. A system was developed specifically for the remote experts to use for telemedical support. Both systems were fully integrated. The integrated system was used and tested in simulated scenarios. It is not clear, however, whether the video streaming was HIPAA compliant. |
|  | Ponce et al. [29] | The smart glass system (Google Glass) used on the local site was a commercial product which had been fully developed and tested by the manufacture. The system used on the remote site was developed with augmented reality features. Both systems were fully integrated. Videostreaming was established by a commercial product (Skype). The integrated system was used and tested with only one patient. Limited information was provided regarding some criteria of TRL 9, such as whether the system had been fully integrated with other operational hardware/software systems (e.g., database), whether all system documentation had been completed, and whether sustaining engineering support team was in place. Finally, it is unclear whether the videostreaming software used in the study complied with HIPAA regulations. |
|  | Demir et al. [31] | The smart glass system used on the local site (Intel Recon Jet) was a commercial product which had been fully developed and tested by its manufacture. In addition, the smart glass system was fully integrated with other operational and network devices (e.g., a mobile router). A specific, dashboard-like system was iteratively designed and developed for the remote site, consisting of several features, such as video/audio call, map view, and annotation). Both systems were fully integrated. The integrated system was used and tested in simulated scenarios. |
|  | Brewer et al. [33] | Both remote providers (trainer) and local providers (learners) wore Google Glasses for surgical training. Video was streamed from the learner's class unit in real time to that of the trainer. The system was tested by 11 surgical residents in a simulated operative field. |
|  | Chai et al. [27] | The smart glass system used on the local site (Google Glass) was a commercial product which had been fully developed and tested by its manufacture. The system used on the remote site (a desktop application with HIPAA compliant text message feature) was fully functional. Both systems were fully integrated. Videostreaming was established by a HIPAA compliant commercial product (Pristine). The integrated system was used and tested with a set of real patient scenarios (n = 18). Remote experts found the quality of audio and visual transmission usable in 16 out of all patient cases. The success rate is 89%. However, limited information was provided regarding some criteria of TRL 9, such as whether the system had been fully integrated with other operational hardware/software systems (e.g., database, hospital IT infrastructure), whether all system documentation had been completed, and whether sustaining engineering support team was in place. |
|  | Noorian et al. [28] | The smart glass system used on the local site (Google Glass) was a commercial product which had been fully developed and tested by their manufactures. The system used on the remote site (an online platform) was also fully functional. Both systems were fully integrated. Videostreaming was established by a HIPAA compliant commercial product (XpertEye). The integrated system was used and tested with a set of real patient scenarios (n = 17). The system was found useful and applicable in the prehospital setting. However, limited information was provided regarding some criteria of TRL 9, such as whether the system had been fully integrated with other operational hardware/software systems (e.g., database, hospital IT infrastructure), whether all system documentation had been completed, and whether sustaining engineering support team was in place. |
| **TRL** | **Study** | **Reasoning/Justification** |
| TRL 8 (n=15) | McCullough et al. [35] | The smart glass system used on the local site (Google Glass) was a commercial product which had been fully developed and tested by the manufacture. The system used on the remote site (a web portal) was also functional. All hardware and software requirements were ensured. Both systems were fully integrated. Videostreaming was established by a HIPAA compliant commercial product (XpertEye). The integrated system was used and tested in 12 bimonthly surgical proctoring sessions held over the course of a 6-month period. However, limited information was provided regarding some criteria of TRL 9, such as whether the system had been fully integrated with other operational hardware/software systems (e.g., database, hospital IT infrastructure), whether all system documentation had been completed, and whether sustaining engineering support team was in place. |
|  | Gupta et al. [30] | The smart glass system (Google Glass) used on the local site was a commercial product which had been fully developed and tested by the manufacture. The system used on the remote site (a web portal) was also functional. Both systems were fully integrated. Videostreaming was established by a HIPAA compliant commercial product. The integrated system was used and tested with a set of real patients (n = 45). However, limited information was provided regarding some criteria of TRL 9, such as whether the system had been fully integrated with other operational hardware/software systems (e.g., database, hospital IT infrastructure), whether all system documentation had been completed, and whether sustaining engineering support team was in place. |
|  | Ho et al. [32] | The smart glass system (Pivothead) used on the local site was a commercial product which had been fully developed and tested by the manufacture. Videostreaming was established by a commercial product (Polycom). The integrated system was used and tested with 37 patients. However, limited information was provided regarding some criteria of TRL 9, such as whether the system had been fully integreated with other operational hardware/software systems (e.g., database, hospital IT infrastructure), whether all system documentation had been completed, and whether sustaining engineering support team was in place. |
|  | Martínez-Galdámez et al. [37] | The smart glass system used on the local site (Iristick) was a commercial product which had been fully developed and tested by its manufacture. The remote expert connects through the application using its own computer. Both systems were fully integrated. The integrated system was used and tested on 2 invitro and 6 real-life cases. Smart glasses were found to be comfortable to wear, and highly accepted by medical providers. However, how video and audio connections were established and whether they were HIPAA compliant were not elaborated in the article. In addition, limited information was provided regarding some criteria of TRL 9, such as whether the system had been fully integrated with other operational hardware/software systems (e.g., database, hospital IT infrastructure), whether all system documentation had been completed, and whether sustaining engineering support team was in place. |
|  | Munusamy et al. [43] | The smart glass system used on the local site (Vuzix M400) was a commercial product which had been fully developed and tested by its manufacture. The remote expert connects through the application via any mobile device. Both systems were fully integrated. The integrated system was used and tested on 102 neurocritical care patients. The system had wide acceptance and high user satisfaction. However, how video and audio connections were established and whether they were HIPAA compliant were not elaborated in the article. In addition, limited information was provided regarding some criteria of TRL 9, such as whether the system had been fully integrated with other operational hardware/software systems (e.g., database, hospital IT infrastructure), whether all system documentation had been completed, and whether sustaining engineering support team was in place. |
| **TRL** | **Study** | **Reasoning/Justification** |
| TRL 8 (n=15) | Datta et al. [36] | The smart glass system used on the local site (Google Glass) was a commercial product which had been fully developed and tested by its manufacture. Videostreaming was enabled by Livestream, which allowed remote experts to observe and to comment within a web-based forum via real-time text or multimedia content posting. The system was used by remote experts and local surgeon trainees (who were in different countries) in 10 surgical operations. However, limited information was provided regarding some criteria of TRL 9, such as whether the system had been fully integrated with other operational hardware/software systems (e.g., database, hospital IT infrastructure), whether all system documentation had been completed, and whether sustaining engineering support team was in place. |
|  | Yoon et al. [44] | The smart glass system used on the local site (Google Glass) was a commercial product which had been fully developed and tested by its manufacture. The remote expert used a desktop application to support the smart glass wearer's work. Both systems were fully integrated. Videostreaming was enabled by APP-RTC. The integrated system was used by 31 medical providers in simulations. The article highlighted a set of issues and barriers in effective use of the system, including unresponsive screen, unstable network, and motion blur. Thus, the system still needs significant improvement. In addition, limited information was provided regarding some criteria of TRL 9, such as whether the system had been fully integrated with other operational hardware/software systems (e.g., database, hospital IT infrastructure), whether all system documentation had been completed, and whether sustaining engineering support team was in place. |
| TRL 9 (n=1) | Diaka et al. [45] | The whole project consisted of not only technology components (e.g., smart glasses, mobile applications), but also care delivery models (e.g., moto-ambulances to facilitate referrals, and upgraded point-of-care tests). The project was running in rural areas of the Democratic Republic of the Congo for over a year, with supporting infrastructure and staff. All stakeholders involved highly valued the intervention. |
